# Senolytic effects of quercetin in an in vitro model of pre-adipocytes and adipocytes induced senescence

**DOI:** 10.1038/s41598-021-02544-0

**Published:** 2021-12-01

**Authors:** Elena Zoico, Nicole Nori, Elena Darra, Maela Tebon, Vanni Rizzatti, Gabriella Policastro, Annamaria De Caro, Andrea Petronio Rossi, Francesco Fantin, Mauro Zamboni

**Affiliations:** 1 Division of Geriatric Medicine and Clinical Nutrition, Department of Medicine, University of Verona, P.le Stefani, 1, 37126 Verona, Italy; 2 Department of Surgery, Dentistry, Pediatrics and Gynecology, University of Verona, Verona, Italy

**Keywords:** Preclinical research, Diseases, Medical research

## Abstract

The dysfunction of adipose tissue with aging and the accumulation of senescent cells has been implicated in the pathophysiology of chronic diseases. Recently interventions capable of reducing the burden of senescent cells and in particular the identification of a new class of drugs termed senolytics have been object of extensive investigation. We used an in vitro model of induced senescence by treating both pre-adipocytes as well as mature adipocytes with hydrogen peroxide (H_2_O_2_) at a sub-lethal concentration for 3 h for three consecutive days, and hereafter with 20 uM quercetin at a dose that in preliminary experiments resulted to be senolytic without cytotoxicity. H_2_O_2_ treated pre-adipocytes and adipocytes showed typical senescence-associated features including increased beta-galactosidase activity (SA-ß-gal) and p21, activation of ROS and increased expression of pro-inflammatory cytokines. The treatment with quercetin in senescent pre-adipocytes and adipocytes was associated to a significant decrease in the number of the SA-β-gal positive cells along with the suppression of ROS and of inflammatory cytokines. Besides, quercetin treatment decreased miR-155-5p expression in both models, with down-regulation of p65 and a trend toward an up-regulation of SIRT-1 in complete cell extracts. The senolytic compound quercetin could affect AT ageing by reducing senescence, induced in our in vitro model by oxidative stress. The downregulation of miRNA-155-5p, possibly through the modulation of NF-κB and SIRT-1, could have a key role in the effects of quercetin on both pre-adipocytes and adipocytes.

## Introduction

Ageing represents one of the major risk factors for the development of chronic diseases and geriatric syndromes, and may increase the risk of death, frailty and hospitalization^1,2^. Aging is characterized by the accumulation of senescent cells in tissues, with an irreversible stop in the replicative cycle, resistance to apoptosis, and an altered pattern of molecules secretion^3–5^.

Adipose tissue (AT) has been recognized as an endocrine organ capable of secreting several bioactive molecules, known as adipokines, playing a crucial role not only in the regulation of energy homeostasis but also in the pathophysiology of insulin resistance, metabolic syndrome and cardiovascular diseases with an increase in their morbidity and the mortality^6,7^. Thus the AT dysfunction, that occurs with aging, has relevant systemic effects, influencing insulin sensitivity, systemic inflammation and ectopic lipid deposition^7^.

The mechanisms that lead to the dysfunction of AT during aging had been object of intense investigation in the last years^3–5,7^. Pre-adipocytes become dysfunctional with aging with a reduced capacity for proliferation and differentiation, leading to an impaired fatty acid handling^8,9^. These dysfunctional pre-adipocytes assume the phenotype of mesenchymal adipocyte-like default cells (MAD)^8^, producing pro-inflammatory cytokines, like TNF-α, involved in the onset of inflammation of aged AT^10,11^. Moreover a variety of endogenous stimuli, as increased oxidative stress and hypoxia, as well as excess nutritional elements such as fatty acids, or products of cell death, may trigger sterile inflammation in AT that is considered to be a major contributor to the chronic low-grade inflammation typical of aging^4,5^. In fact oxidative stress^12,13^ and hypoxia^13^ represent other mechanisms proposed in the dysfunction of the aged AT. The oxidative stress is featured by the excess of radical oxygen species (ROS) which damage lipids, proteins, DNA and mitochondria^14^. Hypoxia might also take a role in cellular senescence, contributing to the production of ROS^13,15^. Finally AT is a site of important accumulation of senescent cells that can secrete a multitude of cytokines, growth factors and matrix metalloproteinases, representing the senescence-associated secretory phenotype (SASP)^4,5^. Recently the differential expression of a particular class of gene regulatory elements, the microRNAs (miRNAs), has been studied in different aged tissues and has also been hypothesized to be involved in the dysfunction of AT^7,16,17^.

In particular miR-155-5p has been recently implicated in several aspects of AT physiology, promoting the differentiation of adipose precursor cells toward white, rather than brown/beige adipocytes, controlling lipolysis and adipocytes’ energy storage and finally activating pro-inflammatory pathways^18^. MiR-155-5p knock-out mice fed with high-calorie diet were protected from obesity, maintained the physiological response to insulin and presented a reduced systemic response to inflammation^19^. In particular the miR-155-5p was associated to the strengthening of inflammation linked to oxidative stress in a process mediated by TNF-α as further described by in vitro experiments^20^. However only a few studies have tried to define the miRNAs expression profile of aged AT and/or upon stress conditions such as oxidative damage: the AT miRNAs profile could be dysregulated, mediating some ageing-associated alterations.

The knowledge of the mechanisms underlying AT aging could lead to the development of treatments aimed at preventing the onset of chronic conditions, maximizing the healthy life span^3,5,7^. Some pharmacologic compounds, called senolytics, have been developed in the last years or investigated for their potential to selectively clear senescent cells in different tissues^3,5^. To date, senolytic activity has been demonstrated for some compounds, mostly in the oncological context^3,5,21^ and only for some tissues^3,5,22^. Only a few compounds have been tested since now for AT^3,5^ and, among those, the polyphenol quercetin^21^ has been shown to have a senolytic effect on AT in in vitro and in vivo experiments^23–26^. However, to our knowledge, there are only a few studies in the literature about the effect of this compound on aged pre-adipocytes and adipocytes and mechanisms of actions are still to be completely defined.

Therefore, the aim of this study was to investigate the effects of quercetin in an in vitro model of induced senescence in pre-adipocytes and adipocytes, subjected to repeated treatments with hydrogen peroxide (H_2_O_2_).

## Methods

### Cell cultures and conditions of treatments

**Figure 1:**
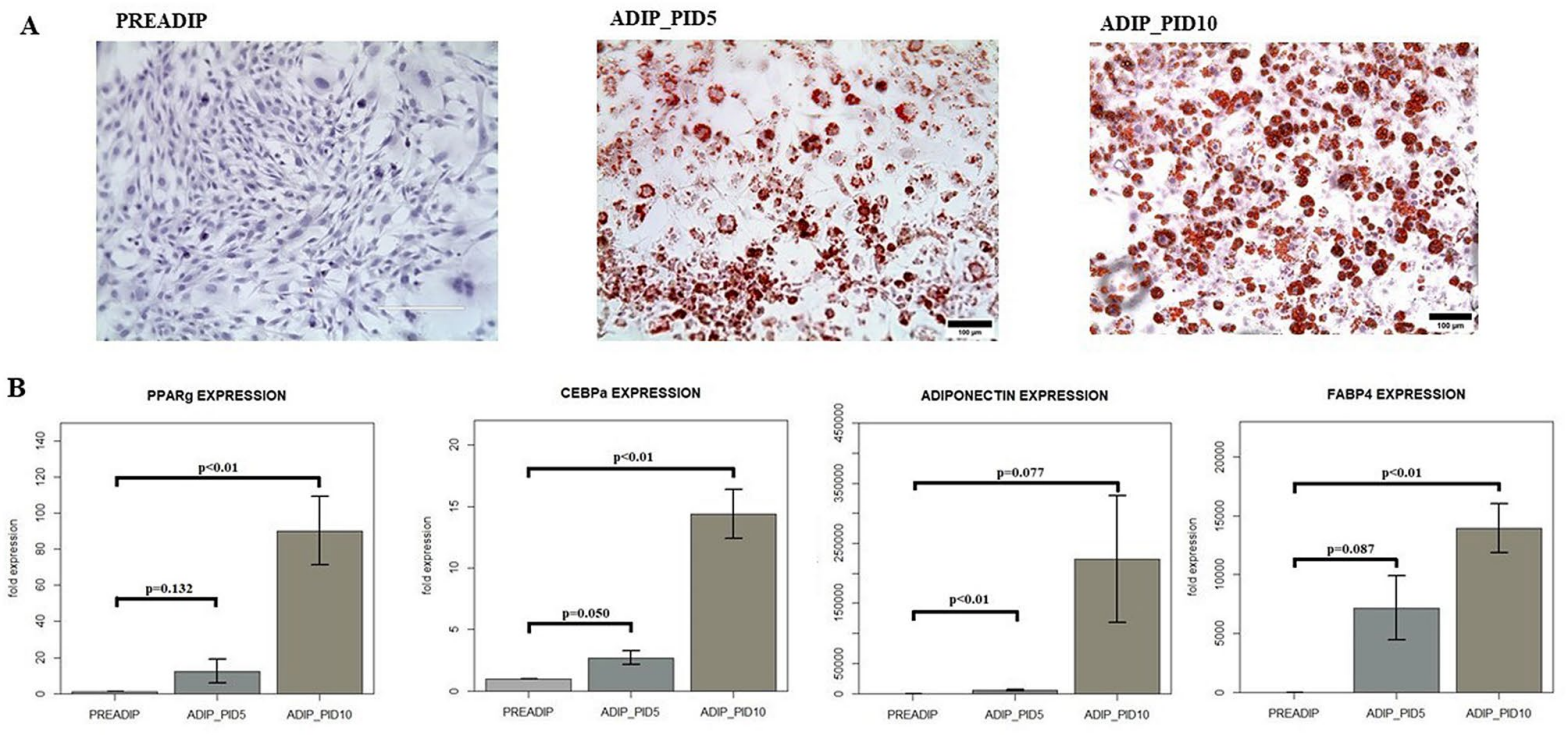
(**A**) Representative images of pre-adipocytes (PREADIP), adipocytes at PID5 (ADIP_PID5) and adipocytes at PID10 (ADIP_PID10), (images acquired by Inverted Laboratory Microscope Leica DM after Oil Red O and Hematoxylin staining—scale bar = 100 μm). (**B**) Gene expression profile of 3T3-L1 at different stages of differentiation. Comparison of gene expression of peroxisome proliferator-activated receptor gamma (PPAR-γ), CCAAT/enhancer-binding protein alpha (CEBP-α), adiponectin and Fatty Acid-Binding Protein 4 (FABP4) between pre-adipocytes (PREADIP), adipocytes at PID5 (ADIP_PID5) and adipocytes at PID10 (ADIP_PID10). PID = post induction day. The values are represented as mean ± SE.

3T3-L1 cells (ECACC Sigma-Aldrich) were cultured at 37 °C in 5% CO2 in DMEM/GlutaMAX culture medium (Gibco), containing FBS 10% and 1% Antibiotic Antimycotic Solution (SIGMA). At 85–90% of confluence, 3T3-L1 cells were detached by trypsin/EDTA (Gibco) and seeded in six wells (Becton Dickinson) containing a pre-sterilized slide (Menzel-Glaser Thermo Scientific). At confluence, cells were induced to differentiate in DMEM/F12 containing 10% FBS, 1% Antibiotic Antimycotic Solution, 0.2 mM IBMX, 10 μM rosiglitazone, 1 μM dexamethasone, 1,72 μM insulin for 3 days. After 72 h post-induction day (post induction day = PID) medium was replaced with an adipocyte maintaining medium (AMM), composed of DMEM/F12 enriched with 10% FBS, 1% Antibiotic Antimycotic Solution, 1.72 μM insulin (Sigma), in which cells were cultured for 2 days (PID5). At PID5, cells were cultured in adipocyte maintaining medium containing DMNEN/F12, 10% FBS, and 1% Antibiotic Antimycotic Solution, which was replaced every 2 days. We considered adipocytes at PID10 as mature adipocytes as cells showed a progressive increase in their area and lipid accumulation as shown after Oil Red O staining (Fig. 1A) and based on previous reports on the morphological and functional changes in adipocytes in the different stages of growth and aging^27–29^. Moreover, to further characterize adipocytes in cultures, we performed gene expression analyses by RT-PCR of key transcriptional factors involved in adipogenesis as well as of key adipokines typical of mature adipocytes (Fig. 1B).

As a model of induced senescence we used a protocol of repeated exposure to hydrogen peroxide (H_2_O_2_) at a sub-lethal concentration (150 μM) (30% w/w in H2O; Sigma) (150 μM) for 3 h for three consecutive days starting from pre-adipocytes at PID 2^30^; at the end of the 3 h, each well was washed twice with PBS and added with complete medium. The cells were collected for analysis after 3 days from the end of the third treatment and adipocytes were then analyzed at PID 8.

Similarly, the adipocytes were stimulated with the same protocol of induced senescence, with H_2_O_2_ (150 μM) for 3 h for three consecutive days, starting from PID 5; the cells were collected for analysis after 3 days from the end of the last treatment at PID 10.

As senolytic treatment, we chose 20 uM quercetin as it was shown as the lowest dose associated to senolytic effects on 3T3-L1 pre-adipocytes and adipocytes^31^ without the cytotoxic effects described for exposure to higher doses^32,33^, in experiments with a time course similar to ours model. Moreover we performed a viability assay by MTT that confirmed unchanged vitality in 20 uM quercetin treated cells compared to controls (Supplementary Fig. 1).

Thus, in a preliminary experiment, we exposed mature adipocytes (PID5) to 20 μM quercetin (CAS-N: 6151–25-3) dissolved in dimethyl sulfoxide (DMSO) at a final concentration of 0.1%, every 48 h to evaluate its antioxidant and senolytic activity; adipocytes were then analyzed at PID 10.

After preliminary data, confirming the senolytic effects of 20 uM quercetin on adipocytes without significant cytotoxicity, we established further experiments where senescence induced 3T3-L1 cells were treated with quercetin. To this porpoise, quercetin 20 μM was added after each treatment with H_2_O_2_ for three consecutive days, in both pre-adipocytes and adipocytes cultures; at the end of the 3 h of H_2_O_2_ treatment, each well was washed twice with PBS before adding quercetin. The cells were collected for analyses after 3 days starting from the end of the last treatment.

### Cytotoxicity assay

As previously shown, H_2_O_2_ treatments can induce cell senescence and oxidative damage without significant cytotoxic effects on 3T3-L1 adipocytes^30^.

The cytotoxicity of the different treatments was determined using the 3-(4,5-dimethylthiazol-2-yl)-2,5-diphenyltetrazolium bromide (MTT) cell proliferation assay kit (Abcam, Cambridge, England) according to the manufacturer's instructions. Briefly, 3T3-L1 pre-adipocytes in the different experimental conditions were incubated with 50 µL of MTT Reagent and 50 µL of serum-free media for each well for 3 h at 37 °C. After incubation, the medium supplemented with the MTT reagent was removed and 150 µl of MTT solvent was added to each well. The absorbance that proportionally indicated the number of viable cells was measured at 590 nm using a PerkinElmer's Victor X4 microplate reader. The cell viability (%) was calculated as 100 × (absorbance of sample-treated cells / absorbance of the control cells)^34^.

The MTT assay confirmed unchanged vitality in H_2_O_2_ treated pre-adipocytes compared to controls (see Supplementary Fig. 1).

### Adipocyte morphology evaluation

After washing with phosphate buffer sulphate (PBS) 0.1 M pH 7.4, cell cultures were fixed for ten min with 10% neutral buffered formalin as previously described^28,29^. Cells were washed with sterile double-distilled water and subsequently stained with a filtered ready to use Oil Red O solution (Bio-Optica, Milano, Italy) for twenty min at room temperature. The cells were then washed with sterile double-distilled water and stained with Mayer's Hematoxylin (Bio-Optica, Milano, Italy) ready-to-use solution for 1 min at room temperature and then washed again with sterile double-distilled water. Slides were treated with Aqueous Mount Quick Medium (Bio-Optica, Milano, Italy). Cells were observed in an EVOS FL Auto Cell Imaging System with EVOS Onstage Incubator (Thermo Fisher Scientific, USA) photo microscope, at 200 × and 400 × magnification. The images were analyzed using ImageJ software 1.51n version (NIH, Bethesda, MD, USA) to count the cells within ten representative fields (200 × magnification, number of cells expressed on mm2) and to calculate the area of fifty randomly chosen cells (200 × magnification; area expressed in μm2).

### Senescence-associated β-galactosidase (SA-β-gal) staining

Senescent cells were detected by using a Senescence Detection Kit (Abcam, Cambridge) as previously described^28^. Cells were cultured in a six-well plate directly on a coverslip, and after the end of the specific treatment period, cells were washed once with 2 ml of 1 × PBS and fixed with 1 ml fixative solution for 10 min at room temperature. Cells were washed twice with 2 ml of 1 × PBS and 1 ml of SA-β-gal staining Solution was added to the plate and incubated for 5 h at 37 °C protected from light. To avoid any effect from the CO_2_, we put the plate inside a Ziplock resealable bag. Cells were then washed once with 1 ml of 1 × PBS and were counterstained with ready-to-use nuclear fast for forty seconds. Cells were observed in an EVOS FL Auto Cell Imaging System with EVOS Onstage Incubator (Thermo Fisher Scientific, USA) photo microscope at 200 × magnification. The images were analyzed using ImageJ software 1.51n (NIH, Bethesda, MD, USA). SA-β-gal-positive cells were quantified by counting stained and unstained cells on 10 random chosen SA-β-gal-positive fields (200 × magnification) and expressed as the per cent of SA-β-gal positive cells over the total counted^35^.

### Analysis of ROS

Intracellular ROS generation was detected by using the Image-iTTM LIVE Green Reactive Oxygen Species Detection Kit (Thermo Fisher Scientific, USA) as previously described^28,29^. Once the cells were treated according to the experimental conditions, cells were washed once with 1 ml of warm HBSS/Ca/Mg and were incubated with 25 μM carboxy-H2DCFDA working solution for 30 min at 37 °C, protected from light. Hoechst 33,342 was added at a final concentration of 1.0 μM to the carboxy- H2DCFDA staining solution during the last 5 min of the incubation. Subsequently, the cells were washed gently three times in warm HBSS/ Ca/Mg, and the coverslip was mounted with Antifade Mounting Medium—Prolong Fluorescence (Boster Biological Technology, USA). Cells were observed in an EVOS FL Auto Cell Imaging System with EVOS Onstage Incubator (Thermo Fisher Scientific, USA) photomicroscope, at 200 × and 400 × magnification. The images were analyzed using ImageJ software 1.51n version (NIH, Bethesda, MD, USA): the mean fluorescence intensity for treatment was calculated on 50 cells positive for ROS at 200 × magnification, computing the integrated density (ID). The background was obtained by measuring the fluorescence intensity of regions outside the cells. The corrected total cell fluorescence (CTCF) was then determined by subtracting the background from the ID (CTCF = ID − background)^28,29^.

### RNA extraction, cDNA synthesis and real-time polymerase chain reaction (RT-PCR)

Total RNA was extracted using the miRNeasy Mini Kit (Qiagen) as previously described^28,29^. Adipocytes were first lysed by Qiashredder column (Qiagen). The cDNA retro-transcription reaction was carried out using the First Strand cDNA Synthesis Kit (Origen) using the following protocol: 5 min at 22 °C, 30 min at 42 °C and 5 min at 85 °C in an Ep Gradient S thermal cycler thermal cycler (Eppendorf). The retro-transcription reaction of the miRNA was performed using the TaqMan MicroRNA Reverse Transcription Kit using 10 ng of total RNA and the specific probe for the miR-155-5p and for the U6 snRNA normalizer (Termofisher Scientific) with the following protocol: 30 min at 16 °C, 30 min at 42 °C and 5 min at 85 °C. The amplification reaction of the mRNA was carried out with the CFX96 Real-Time machine using the SsoAdvanced Universal SYBR Green Supermix (Bio-Rad), as per the guide. Briefly, the RT-PCR reaction was performed in a total volume of 20 μl by amplifying 50 ng of cDNA for 40 cycles under the following experimental conditions: 5 s at 95 °C and 30 s at 60 °C. Pairs of sequence-specific primers were designed to amplify a small DNA fragment (150 bp) for Peroxisome proliferator activated receptor gamma (PPAR-γ) (FW, 5′-cataaagtccttcccgctga-3′; RV, 5′-gaaactggcacccttgaaaa-3′), CCAAT/enhancer binding protein alpha (CEBPα) (FW, 5′-caagaacagcaacgagta-3′; RV, 5′- ttgaccaaggagctctca-3′), Adiponectin (FW, 5′-ggagatgcaggtcttcttgg-3′; RV, 5′-cgaatgggtacattgggaac-3′), Fatty acid binding protein 4, adipocyte (FABP4) (FW, 5′-acgacaggaaggtgaagagc-3′; RV, 5′-cttgtggaagtcacgccttt-3′), IL-6 (FW, 5′- agacaaagccagagtccttca-3′; RW, 5′-gagcattggaaattggggta-3′), p21 (FW gtgggcacttcagggtttt; RW ttgtcgctgtcttgcactct), MCP-1 (FW- aggtccctgtcatgcttctg; RW- gctgctggtgatcctcttgt). The miRNA amplification reaction was carried out with the CFX96 Real-Time machine using the TaqMan Universal PCR Master Mix (ThermoFisher Scientific), as a guide. Briefly, the RT-PCR reaction was performed in a total volume of 20 μl by amplifying 0.5 ng of specifically retro-transcribed miRNA for 40 cycles under the following experimental conditions: 50 °C 2 min and 95 °C 10 min for l activation of the enzyme and at 95 °C for 15 s and 60 °C for 1 min for forty cycles. The level of mRNA expression was assessed using an internal control gene; for pre-adipocytes the GAPDH gene (FW-aactttggcattgtggaag; RW-acacattgggggtaggaaca) while for mature adipocytes TBP was used (cat. n. 10025636). The relative expression was calculated using the formula 2- (ΔΔCt). Each analysis was conducted at least in duplicate.

### Protein analysis by Western Blot (WB)

The proteins were extracted from cell pellets using Pierce RIPA buffer (product code 89901, Thermofisher) added with the cocktail of protease inhibitors (Roche cat. n. 11697498001). The quantification of total proteins was carried out using the Pierce BCA Protein Assay (ref. 23225, Thermo Fisher Scientific). An overall protein concentration of 25 ng for each sample was loaded in 7.5% SDS gel-acrylamide. The run was performed under the following conditions: 100 V for 90 min using the running buffer at 0.1% of SDS. The proteins were blotted in PVDF membrane (Immobilon P, Millipore, Bedford MA) at constant 100 V. The filter was incubated for 1 h at room temperature, in a blocking solution (BSA, Albumin Bovim Serum, Sigma) 5%, Tris–HCl pH 7.5 10 mM, NaCl 100 mM, Tween 20 0.1%. The membrane was then incubated overnight at 4 °C under stirring, with a primary monoclonal antibody in the blocking solution. The membrane was washed three times for 10 min in a solution containing TBS added to 0.1% Tween 20. The filter was incubated for 1 h under stirring and at room temperature with the secondary antibody conjugated with a peroxidase, diluted in the blocking solution. The filter was rewashed (three times for 10 min) from the excess antibody. The bands on the membrane were detected using chemiluminescence which develops following the enzymatic reaction of the peroxidase in the presence of hydrogen peroxide and luminol. The ECL detection kit ECL Select TM Western Blotting Detection Reagent GE Healthcare was used as the substrate according to the proportions recommended by the supplier. The antigen–antibody complexes that reacted with the substrates were revealed by Bio-Rad ChemiDoc XRS. The antibodies used were NF-κB p65 (D14E12) XP Rabbit mAb 8242, SIRT1 (D1D7) Rabbit mAb 9475, Anti-rabbit IgG, HRP-linked antibody 7074, α tubulin (Cell Signaling). All the WB were done in triplicate: in each figure in the text is provided an explanatory image for all the blots with the mean relative densitometric analysis; all full-length membranes are presented in Supplementary Fig. 2A–D.

### Statistical analysis

Data were presented as means ± standard error (SE) and each experimental condition was performed in triplicate. Variables which did not show a normal distribution were log-transformed before analyses. Differences between groups were evaluated by univariate analysis (ANOVA). A *p*-value < 0.05 was used to determine statistical significance. All statistical analyses were performed using the SPSS statistical package.

## Results

### An in vitro model to study the senolytic effects of quercetin in pre-adipocytes and adipocytes

In order to study the senolytic effects of quercetin in AT, we established an in vitro model of pre-adipocytes as well as mature adipocytes cultures by using 3T3-L1 cells.

3T3-L1 cells were induced and differentiated according to previous reports that described into details the morphological and functional changes of pre-adipocytes and adipocytes in the different stages of growth and aging^27–29^. 3T3-L1 cells during differentiation show significant morphological changes with a progressive increase in lipid accumulation and cell area from preadipocytes to young (PID5) until mature adipocytes (PID 10) (Fig. 1). Moreover gene expression analyses of key transcriptional factors involved in adipogenesis, as well as of some adipokines, confirm the onset of an adipocyte phenotype as shown by the significant increase in PPAR-γ and CEBPα expression from pre-adipocytes to adipocytes at PID 5 and at PID 10 (Fig. 1); similarly also adiponectin and FABP4 expression levels increase progressively from pre-adipocytes until adipocytes at PID10 (Fig. 1).

### The effects of quercetin in mature adipocytes

As senolytic treatment, based on previously published experiments^31–33,36,37^, we chose to test quercetin at 20 uM, as it was shown as the lowest dose associated to potential senolytic effects on 3T3-L1 pre-adipocytes and adipocytes^31,36,37^ without the cytotoxic effects described for exposure to higher doses^32,33^, in experiments with a time course similar to ours models.

Thus, we exposed 3T3-L1 cells 5 days after the induction (at PID5), to quercetin 20 μM, repeating the treatment on alternate days for a total of three times, analyzing the cells at PID10.

**Figure 2:**
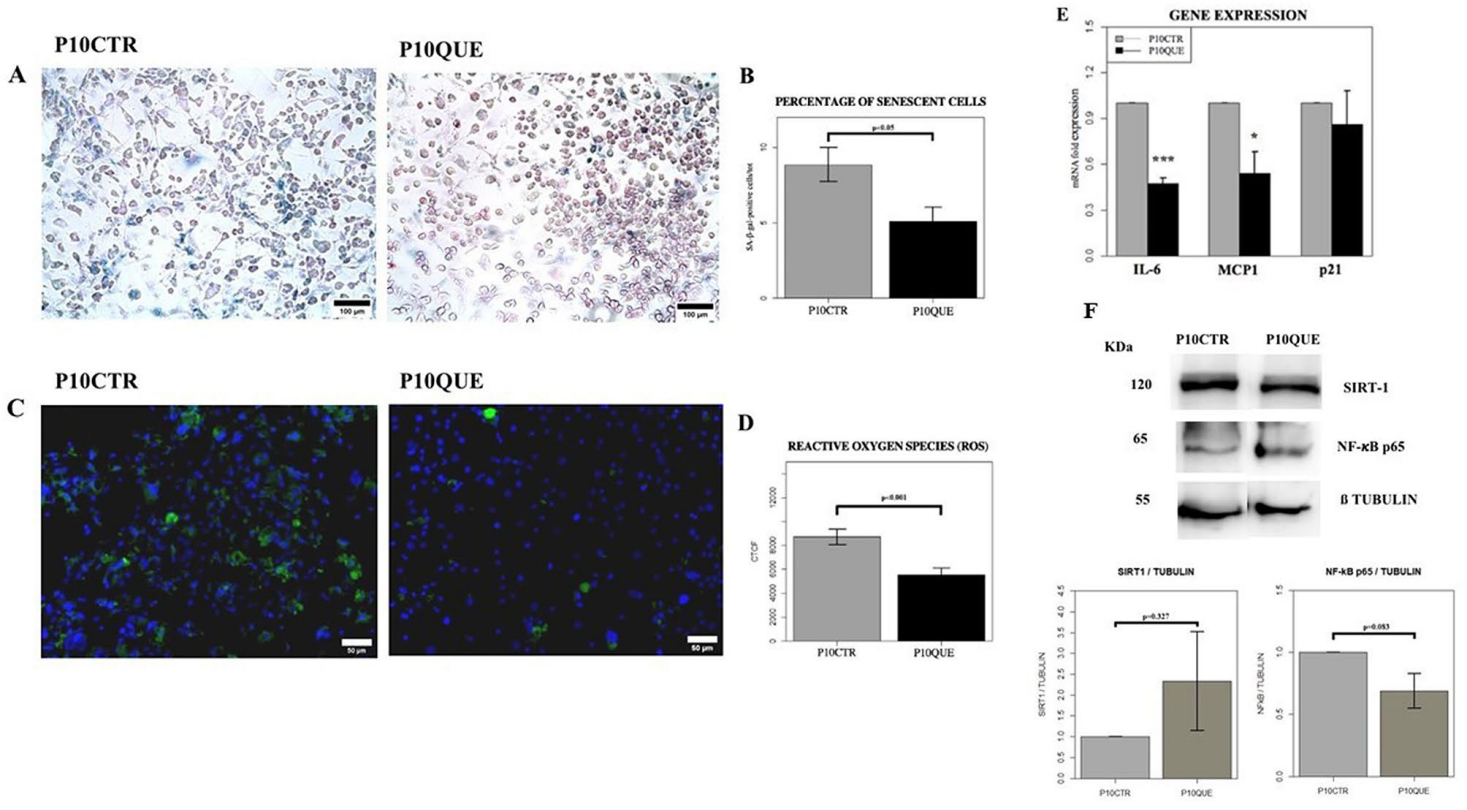
Analyses on 3T3-L1 cells treated with quercetin 20 μM 5 days after the induction (at PID5) on alternate days for a total of three times; cells were analyzed at PID10. (**A**) Evaluation of the adipocyte’s senescence with the specific staining for β-galactosidase. Representative images of cell senescence in adipocytes at PID10 (P10CTR) and PID10 treated with 20 μM quercetin (P10QUE). Scale = 100 μm. (**B**) Histograms showing the percentage of senescent cells among adipocytes (P10CTR) and adipocytes treated with 20 μM quercetin (P10QUE), at the 10th day after induction. The values are represented as mean ± SE. (**C**) Significant images of the accumulation of intracellular ROS (in green) in 3T3-L1 fat cells at PID10 (P10CTR) and PID10 treated with 20 μM quercetin (P10QUER). Scale = 50 μm. (**D**) Histograms showing the amount of ROS among adipocytes (P10CTR) and adipocytes treated with 20 μM quercetin (P10QUE), at the 10th day after induction. The values are represented as mean ± SE. PID = post induction day, ROS = Reactive oxygen species. (**E**) Gene expression profile of adipocytes at PID10 (P10CTR) and adipocytes treated with quercetin (P10QUE) at PID10. *p < 0.05, **p < 0.01, ***p < 0.001. IL-6 = interleukin-6, MCP1 = monocyte chemoattractant protein 1, p21 = cyclin-dependent kinase inhibitor 1. (**F**) Cropped blots and densitometric analysis from western blotting assay of SIRT1 and NF-κB p65 normalized on β-tubulin, in total extracts of adipocytes at PID10 (P10CTR) and adipocytes at PID10 treated with quercetin (P10QUE). The values are represented as mean ± SE. SIRT1 = silent regulator information 1, NF-κB = nuclear factor kappa-light-chain-enhancer of activated B cells. For full length membranes please refer to Supplementary Fig. 2A, B.

Adipocytes treated with quercetin, compared to the controls, showed a significant decline in the expression of the SA-β-gal enzyme (*p* < 0.05) (Fig. 2A, B). After treatment with 20 μM quercetin, adipocytes showed a significant reduction in the intracellular accumulation of ROS compared to untreated controls (*p* < 0.01) (Fig. 2C, D). In addition, at the level of the gene expression, we observed that IL-6 and MCP-1 mRNA were significantly reduced in the adipocytes treated with quercetin compared to the controls (Fig. 2E). The expression of p65 NF-κB, analyzed by WB in complete cell extracts, was reduced in the treated adipocytes compared to the controls, whereas SIRT1 was tendentially increased in adipocytes treated with the flavonoid compared to the controls (Fig. 2F).

### The effects of quercetin on the process of in vitro induced senescence in mature adipocytes

To induce cellular senescence, as s previously described^30^, adipocytes were exposed to a protocol of repeated treatments with H_2_O_2_ at a sub-lethal concentration that was shown to induce senescence, with enhanced SA-β-gal activity and increased p53 and p21 expression; moreover the protocol produced a SASP phenotype, with enhanced gene expression of TNF-alpha, IL-6 and iNOS and of NF-kB, and was associated to oxidative damage as indicated by accumulation of intracellular ROS^30^.

**Figure 3:**
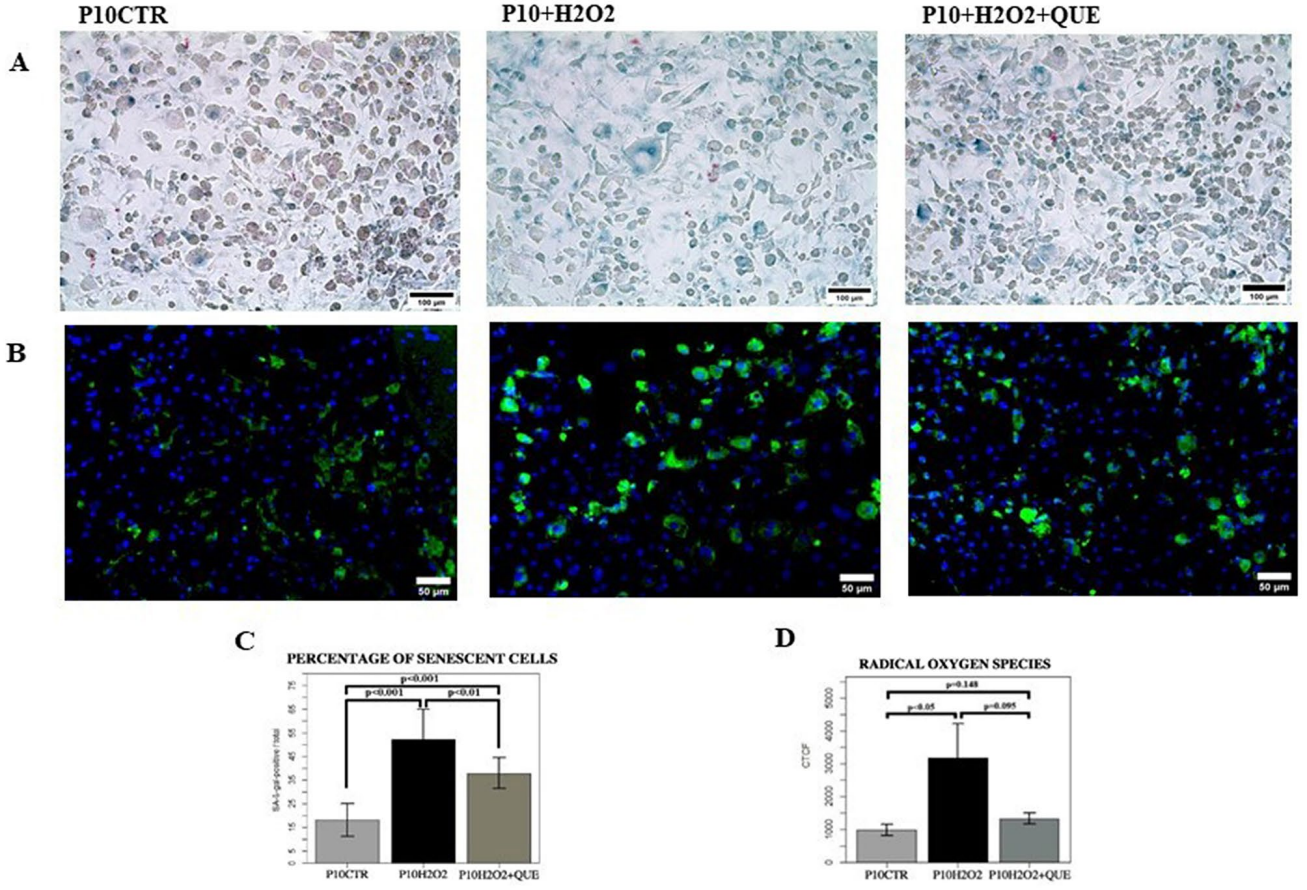
(**A**) Evaluation of the adipocyte’s senescence with the specific staining for β-galactosidase. Representative images of cell senescence in adipocytes at PID10 (P10CTR), adipocytes stimulated with H_2_O_2_ (P10H2O2) and treated with H_2_O_2_ plus quercetin (P10H2O2 + QUE). Scale = 100 μm. (**B**) Significant images of the accumulation of intracellular ROS (in green) in 3T3-L1 fat cells at PID10 (P10CTR), PID10 treated with H_2_O_2_ (P10H2O2) and PID10 treated with H_2_O_2_ plus quercetin (P10H2O2 + QUE). Scale = 50 μm. (**C**) Histograms showing the percentage of senescent cells among adipocytes (P10CTR), adipocytes treated with H_2_O_2_ and adipocytes treated with H_2_O_2_ plus quercetin (P10H2O2 + QUE), at the 10th day after induction. The values are represented as mean ± SE. (**D**) Histograms showing the amount of ROS among adipocytes (P10CTR), adipocytes treated with H_2_O_2_ (P10H2O2) and PID10 treated with H_2_O_2_ plus quercetin (P10H2O2 + QUE), at the 10th day after induction. The values are represented as mean ± SE.

**Figure 4:**
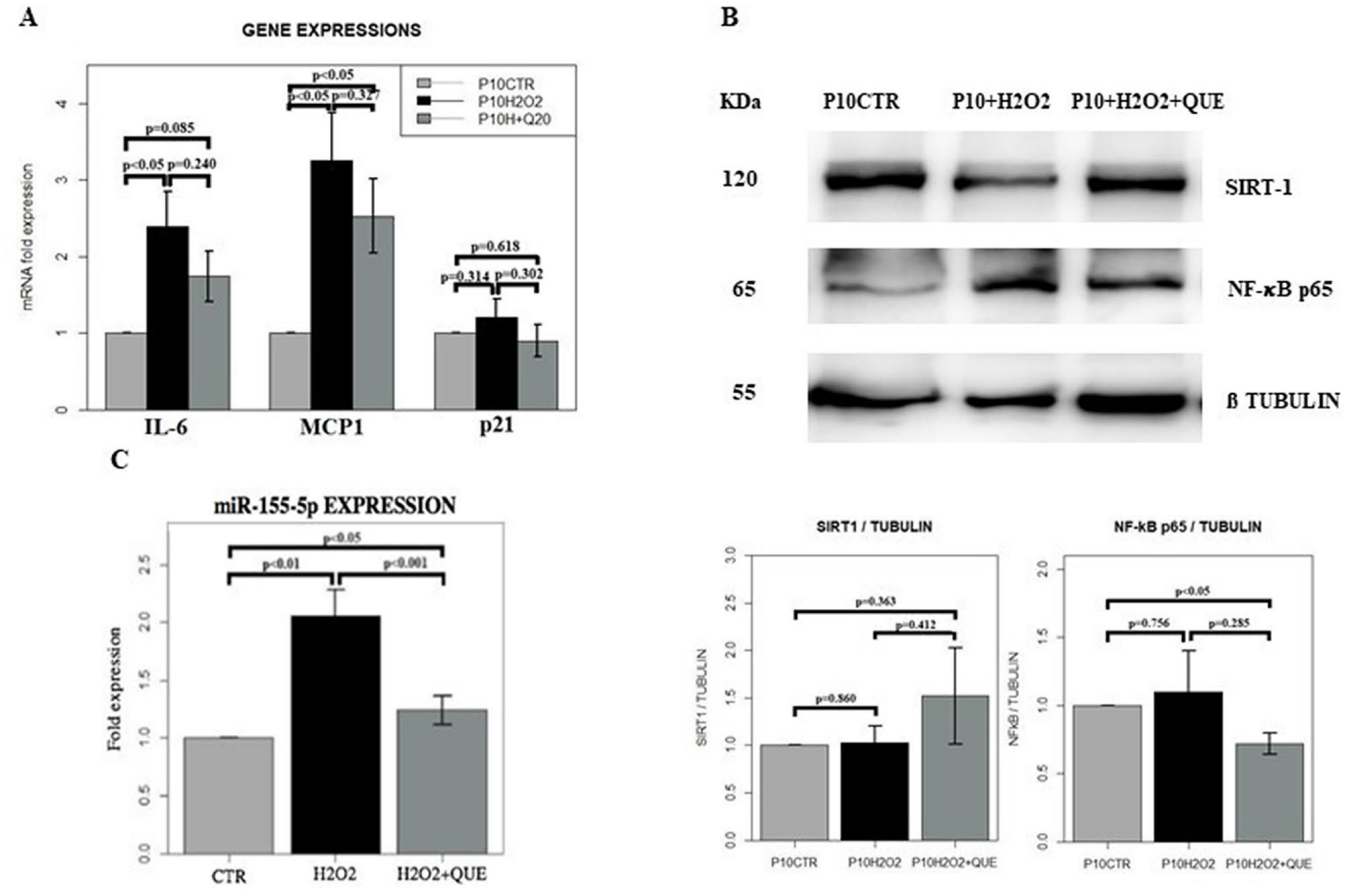
(**A**) Gene expression profile of adipocytes at PID10 (P10CTR), adipocytes at PID10 treated with H_2_O_2_ (P10 + H2O2) and adipocytes at PID10 treated with H_2_O_2_ plus quercetin (P10 + H2O2 + QUE). *p < 0.05, **p < 0.01, ***p < 0.001. IL-6 = interleukin-6, MCP1 = monocyte chemoattractant protein 1, p21 = cyclin-dependent kinase inhibitor 1. (**B**) Cropped blots and densitometric analysis from western blotting assay of SIRT1 and NF-κB p65 normalized on β-tubulin, in adipocytes at PID10 (P10CTR), adipocytes at PID10 treated with H_2_O_2_ (P10 + H2O2) and adipocytes at PID10 treated with H_2_O_2_ plus quercetin (P10 + H2O2 + QUE). The values are represented as mean ± SE. SIRT1 = silent regulator information 1, NF-κB = nuclear factor kappa-light-chain-enhancer of activated B cells. For full length membranes please refer to Supplementary Fig. 2A, B. (**C**) Histograms showing the expression of miR-155-5p in adipocytes at PID10 (CTR), adipocytes at PID10 treated with H_2_O_2_ (H2O2) and adipocytes at PID10 treated with H_2_O_2_ plus quercetin (H2O2 + QUE). SIRT1 = silent regulator information 1, NF-κB = nuclear factor kappa-light-chain-enhancer of activated B cells.

In our experiments, the exposure of mature adipocytes to H_2_O_2_ confirmed to be associated to the induction of senescence, as shown by the significant increase in the number of positive SA-β-gal cells (*p* < 0.01) (Fig. 3A, C) and by the tendential increase in p21 gene expression (Fig. 4A). After H_2_O_2_ treatments there was also a significant increase in intracellular ROS accumulation (*p* < 0.05) (Fig. 3B, D) compared to controls.

In our cultures, mature adipocytes treated with H_2_O_2_ presented likely a SASP phenotype as suggested by the significant increased gene expression of IL-6 and MCP-1 compared to the controls (*p* < 0.05) (Fig. 4A). Interestingly we also observed a significant increase in the expression of miR-155-5p in mature adipocytes treated with H_2_O_2_ compared to controls (*p* < 0.01) (Fig. 4C).

In this model of induced senescence of adipocytes after H_2_O_2_ exposure, we tested the effects of treatment with 20 μM quercetin, a dose associated with a senolytic and anti-inflammatory effect in our preliminary experiment (Fig. 2).

Quercetin treatment significantly decreased the SA-β-gal activity in H_2_O_2_ treated adipocytes (*p* < 0.01) (Fig. 3A, C). Moreover, the intracellular accumulation of ROS in the H_2_O_2_ treated adipocytes, underwent toward a reduction after quercetin exposure (*p* = 0.095) (Fig. 3B, D). After administration of quercetin, there was also a trend toward a decrease in inflammatory genes expression such as IL-6 and MCP-1 compared to H_2_O_2_ treated adipocytes (Fig. 4A). As well, the treatment with quercetin of adipocytes with induced senescence, decreased total expression of p65 and tendentially increased the expression of SIRT 1, as shown by WB analyses (Fig. 4B). Finally, the treatment with quercetin significantly modulated the expression of miR-155-5p in H_2_O_2_ treated adipocytes, by significantly reducing its expression to the levels like those of controls (*p* < 0.001) (Fig. 4C).

### The effects of quercetin on the process of in vitro induced senescence in pre-adipocytes

At the 80% of the confluence, 3T3-L1 pre-adipocytes were stimulated with H_2_O_2_ for 3 h and for three consecutive days; in the co-treated samples, quercetin 20 μM was added after each treatment with H_2_O_2_ for three consecutive days as well. The cells were collected 3 days after the last treatment in order to allow the onset of senescence.

**Figure 5:**
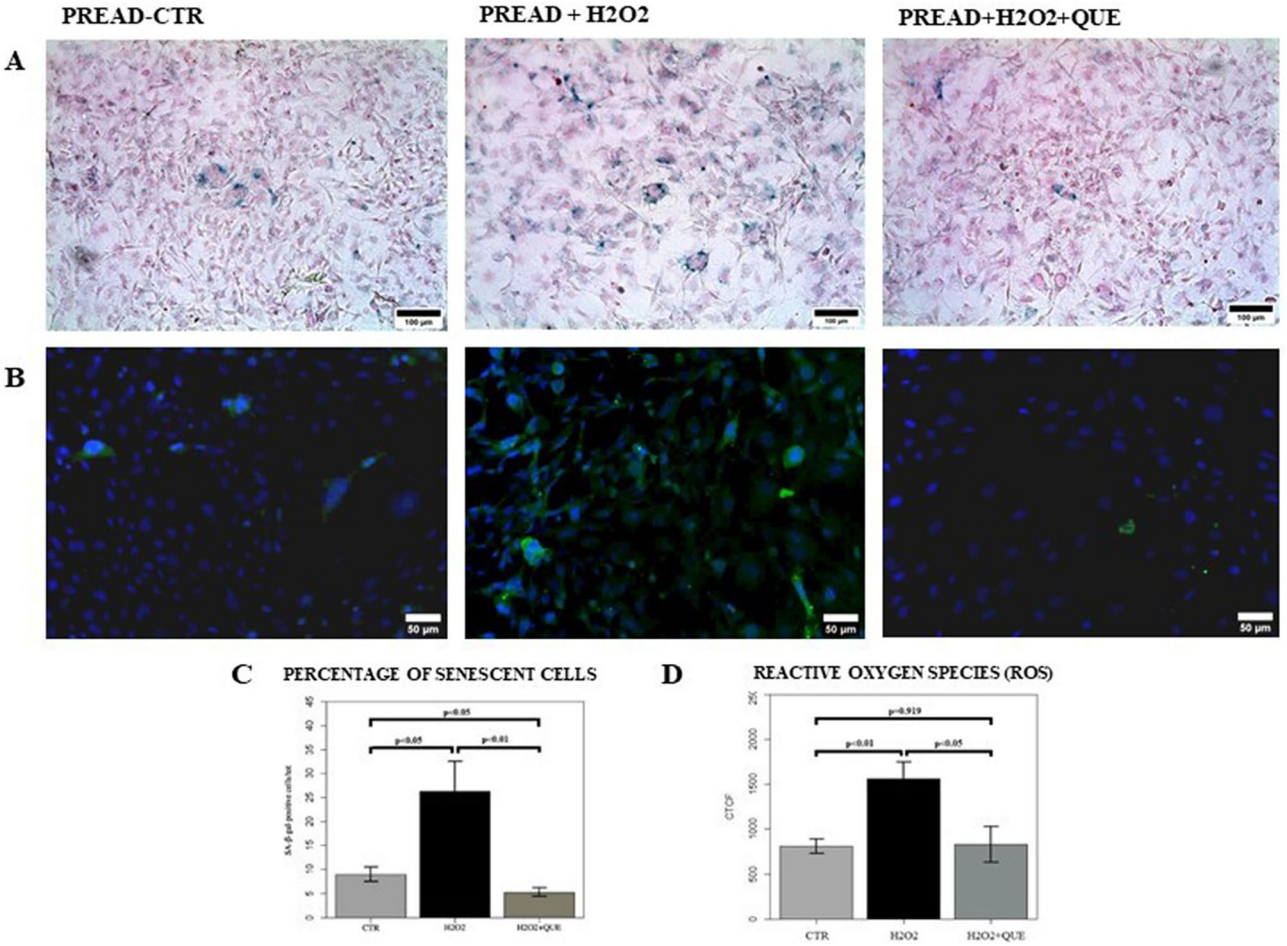
(**A**) Evaluation of the pre-adipocytes senescence with the specific staining for β-galactosidase. Representative images of cell senescence in pre-adipocytes (PREAD-CTR), pre-adipocytes stimulated with H_2_O_2_ (PREAD + H2O2) and treated with H_2_O_2_ plus quercetin (PREAD + H2O2 + QUE). Scale = 100 μm. (**B**) Significant images of the accumulation of intracellular ROS (in green) in pre-adipocytes (PREAD-CTR), pre-adipocytes treated with H_2_O_2_ (PREAD + H2O) and pre-adipocytes treated with H_2_O_2_ plus quercetin (PREAD + H2O2 + QUE). Scale = 50 μm. (**C**) Histograms showing the percentage of senescent cells among pre-adipocytes (CTR), pre-adipocytes treated with H_2_O_2_ (H2O2) and pre-adipocytes treated with H_2_O_2_ plus quercetin (H2O2 + QUE). The values are represented as mean ± SE. (**D**) Histograms showing the amount of ROS among pre-adipocytes (CTR), pre-adipocytes treated with H_2_O_2_ (H2O2) and pre-adipocytes treated with H_2_O_2_ plus quercetin (H2O2 + QUE). The values are represented as mean ± SE. ROS = Reactive oxygen species.

**Figure 6:**
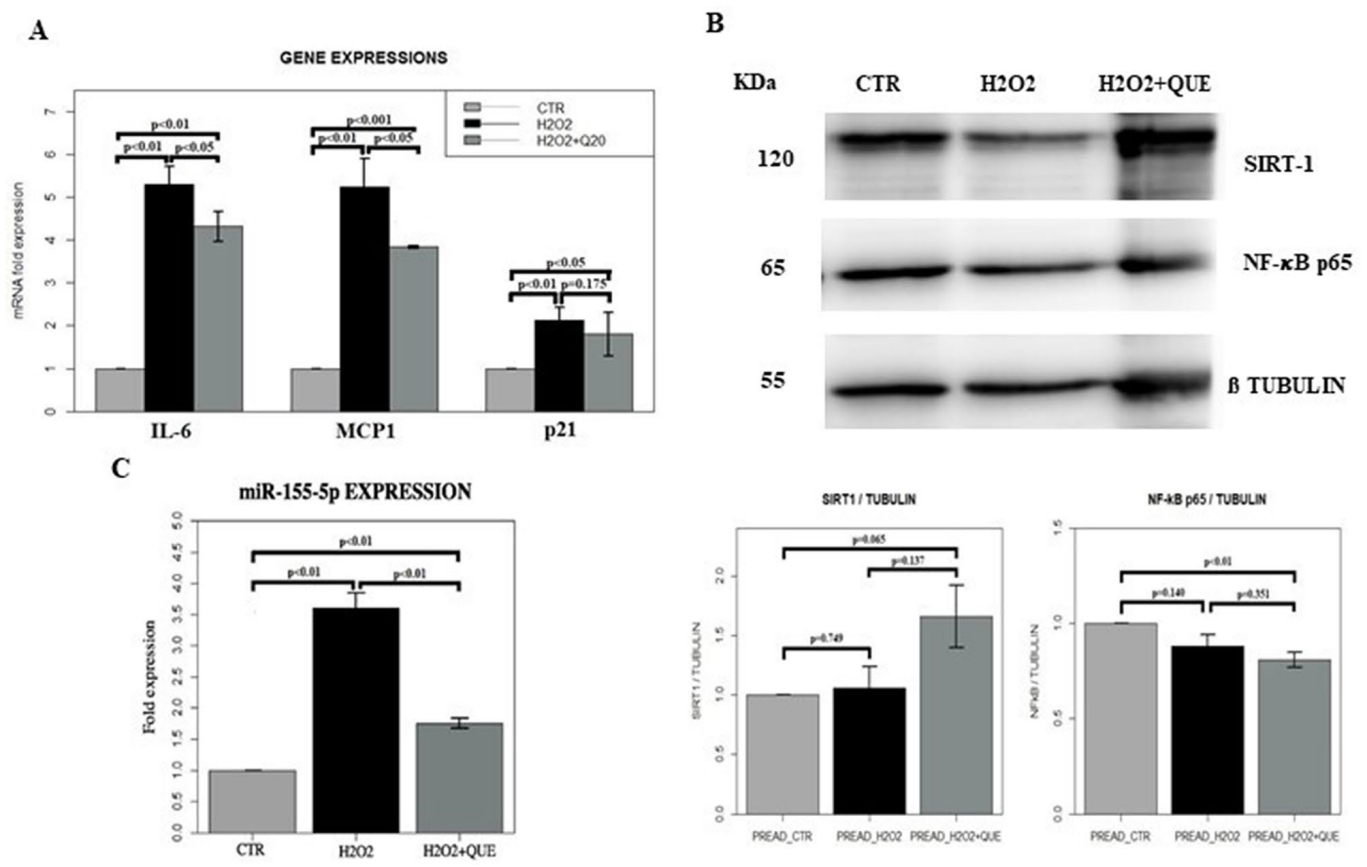
(**A**) Gene expression profile of pre-adipocytes (PREAD-CTR), pre-adipocytes treated with H_2_O_2_ (PREAD-H2O2) and pre-adipocytes treated with H_2_O_2_ plus quercetin (PREAD-H2O2 + QUE). IL-6 = interleukin-6, MCP1 = monocyte chemoattractant protein 1, p21 = cyclin-dependent kinase inhibitor 1. (**B**) Cropped blots and densitometric analysis from western blotting assay of SIRT1 and NF-κB p65 normalized on β-tubulin in pre-adipocytes (CTR), pre-adipocytes treated with H_2_O_2_ (H2O2) and pre-adipocytes treated with H_2_O_2_ plus quercetin (H2O2 + QUE). The values are represented as mean ± SE. SIRT1 = silent regulator information 1, NF-κB = nuclear factor kappa-light-chain-enhancer of activated B cells. For full length membranes please refer to Supplementary Fig. 2C, D. (**C**) Histograms showing the expression of miR-155-5p in pre-adipocytes (CTR), pre-adipocytes treated with H_2_O_2_ (H2O2) and adipocytes treated with H_2_O_2_ plus quercetin (H2O2 + QUE). SIRT1 = silent regulator information 1, NF-κB = nuclear factor kappa-light-chain-enhancer of activated B cells.

As expected 3T3-L1 pre-adipocytes stimulated with H_2_O_2_ presented a significant increase in the expression of the beta-SA-β-gal enzyme (*p* < 0.05) (Fig. 5A, C) as well as in p21 expression (*p* < 0.01) (Fig. 6A). After H_2_O_2_ treatments there was also a significant increase in the production of intracellular ROS compared to untreated pre-adipocytes (*p* < 0.01) (Fig. 5B, D).

In addition, treatment with H_2_O_2_, as previously shown^30^ conferred likely a SASP phenotype to the pre-adipocytes, as suggested by the significant increase in the gene expression of IL-6 and MCP-1 compared to the controls (*p* < 0.01) (Fig. 6A). In addition, we observed a significant increase in the pro-inflammatory miR-155-5p (*p* < 0.01) (Fig. 6C).

In this model of pre-adipocytes induced senescence, obtained after H_2_O_2_ exposure, treatment with 20 μM quercetin significantly reduced the SA-β-gal activity (*p* < 0.01) (Fig. 5A, C). After treatment with the flavonoid, intracellular ROS expression was also significantly reduced (*p* < 0.05) (Fig. 5B, D). At the same time, there was a significant decrease in the expression of the pro-inflammatory cytokines IL-6 (*p* < 0.05) and MCP-1 (*p* < 0.05) in pre-adipocyte treated with H_2_O_2_ and quercetin compared to H_2_O_2_ treated cells (Fig. 6A). In the pre-adipocytes treated with the flavonoid, there was also a modulation of p65 in complete cell extracts, which was reduced after the treatment with quercetin (*p* < 0.01), whilst after treatment we found an increase in SIRT1 (*p* 0.065) (Fig. 6B). Moreover, quercetin modulated the expression of miR-155-5p in H_2_O_2_ treated pre-adipocytes which underwent a significant decrease compared to H_2_O_2_ treated only pre-adipocytes (*p* < 0.01) (Fig. 6C).

## Discussion

Our study shows that quercetin may act as a potential senolytic and anti-inflammatory agent in senescent adipocytes as well as in senescent pre-adipocytes. In our in vitro model of induced-senescence obtained by H_2_O_2_ treatment, both pre-adipocytes and adipocytes showed characteristic senescence-associated features including increased SA-ß-gal activity, ROS activation and increased pro-inflammatory gene. The treatment with quercetin both in H_2_O_2_ treated pre-adipocytes and adipocytes, reduced their senescence associated features, in particular decreasing the number of the SA-β-gal positive cells along with the suppression of ROS and of inflammatory cytokines. Interestingly quercetin was associated to decreased miR-155-5p expression in both models, with down-regulation of the expression of total p65 and a trend toward an up-regulation of SIRT-1.

Translational research has recently pointed the attention on the study of fundamental aging mechanisms, including cellular senescence and progenitor cell dysfunction as they may represent potential therapeutic targets to alleviate multiple age-related diseases^38^ and to delay the aging process itself^39^. A variety of stressors, including oxidative stress, DNA damage and oncogene activation may induce cellular senescence that is essentially an irreversible cell fate, in which cells stop dividing in response to an insult^40^. Strategies for selective elimination of senescent cells are beginning to emerge following the findings of studies conducted on animals^4,7,31^.

Genetic clearance of senescent cells in mice was proved to induce improvement of age related AT dysfunction^7^. Starting from the study by Zhu and colleagues^31^, a new class of drugs, termed senolytics, which selectively kill senescent cells, was discovered. Senolytic agents, selectively clearing senescent cells by inhibition of cellular survival pathways, protect the tissues against the effect of their own SASP^3, 41^.

In this in vitro model, we found that quercetin may counteract senescence of pre-adipocytes and adipocytes, determining in both cells a significantly decreases in SA-β-gal activity, ROS production, and inflammation.

Our findings are in line with recent observations conducted in mice^23,31,41,42^ and with a human study^43^ where quercetin was co administered with other compounds. In mice intermittent administration of dasatinib and quercetin determined a decline in senescent cells and improvement in glucose tolerance, completely abolishing AT inflammation induced by high fat diet (HFD)^42^. Moreover, HFD mice treated with resveratrol and quercetin, had significantly lower body weights compared with the HFD group and had significantly reduced visceral adipose tissue and adipocyte sizes^42^. In humans a 3-day treatment of dasatinib and quercetin declined AT senescent cell burden and macrophage accumulation, enhanced adipocyte progenitor replicative potential, and reduced key circulating SASP^43^.

However, the targets and complex mechanisms of action of quercetin are still not completely known^44^. In general, a senolytic compound targets not only a single receptor, enzyme or biochemical pathway, but several senescent cell antiapoptotic pathways (SCAPs) (as ephrins/dependence receptors; PI3Kd/Akt/metabolic; Bcl-2/B-cell lymphoma-extralarge (Bcl-xl)/Bcl-w; p53/FOXO4a/p21/ serpine [PAI- 1&2]; HIF-1a and the HSP-90 pathway)^3,41^.

Some studies show that quercetin, like other flavonoids, can exert a broad-spectrum of biological activities due to its antioxidant and anti-inflammatory properties^44^. In particular quercetin can inhibit multiple pathways related to inflammation in different tissues as COX-2, nuclear factor-kappa B (NF-κB), activator protein 1 (AP-1), mitogen-activated protein kinase (MAPK), reactive nitric oxide synthase, (NOS) and reactive C-protein (CRP)^44^.

In the literature, quercetin was associated with a down-regulation of the expression of NF-κB and with an up-regulation of SIRT-1; interestingly in our experiment, in both senescent preadipocytes and adipocytes, these effects were paralleled by a decrease in miR-155-5p expression.

To our knowledge no data are available in the literature about quercetin regulation of miR-155 in preadipocytes and adipocytes.

MiR-155-5p has been implicated in enhancing adipogenic differentiation^17^, in lypolisis regulation^17^ and has been considered a positive modulator of inflammation in AT thus influencing AT accumulation in obesity^18,20^.

In fact, miR-155-5p has been identified as a pro-inflammatory mediator in monocytes and macrophages^18^, and an increase in miR-155-5p was already found in human umbilical endothelial cells after treatment with H_2_O_2_^20^.

Adipogenesis is regulated by several adipocyte-selective microRNAs (miRNAs) and transcription factors and the expression of several miRNAs in pre-adipocytes and in adipocytes are significantly changed in obesity^17^. Involvement of miR-155-5p in aging processes, as well as in AT dysfunction has been also confirmed by the observation that miR-155-5p deletion in HFD mice prevented adipocyte hypertrophy and AT inflammation, upregulating adipogenic and glucose metabolism genes^19^. Moreover miR-155 knock-out pre-adipocytes presented in culture an increased differentiation showing an up-regulation of CEBP-ß and of PPAR-γ gene expression^19^.

In our in vitro study quercetin significantly decreased miR-155-5p expression both in pre-adipocytes and adipocytes confirming a previous study conducted in murine macrophages where miR-155 was down-regulated by quercetin and its metabolites^45^.

**Figure 7:**
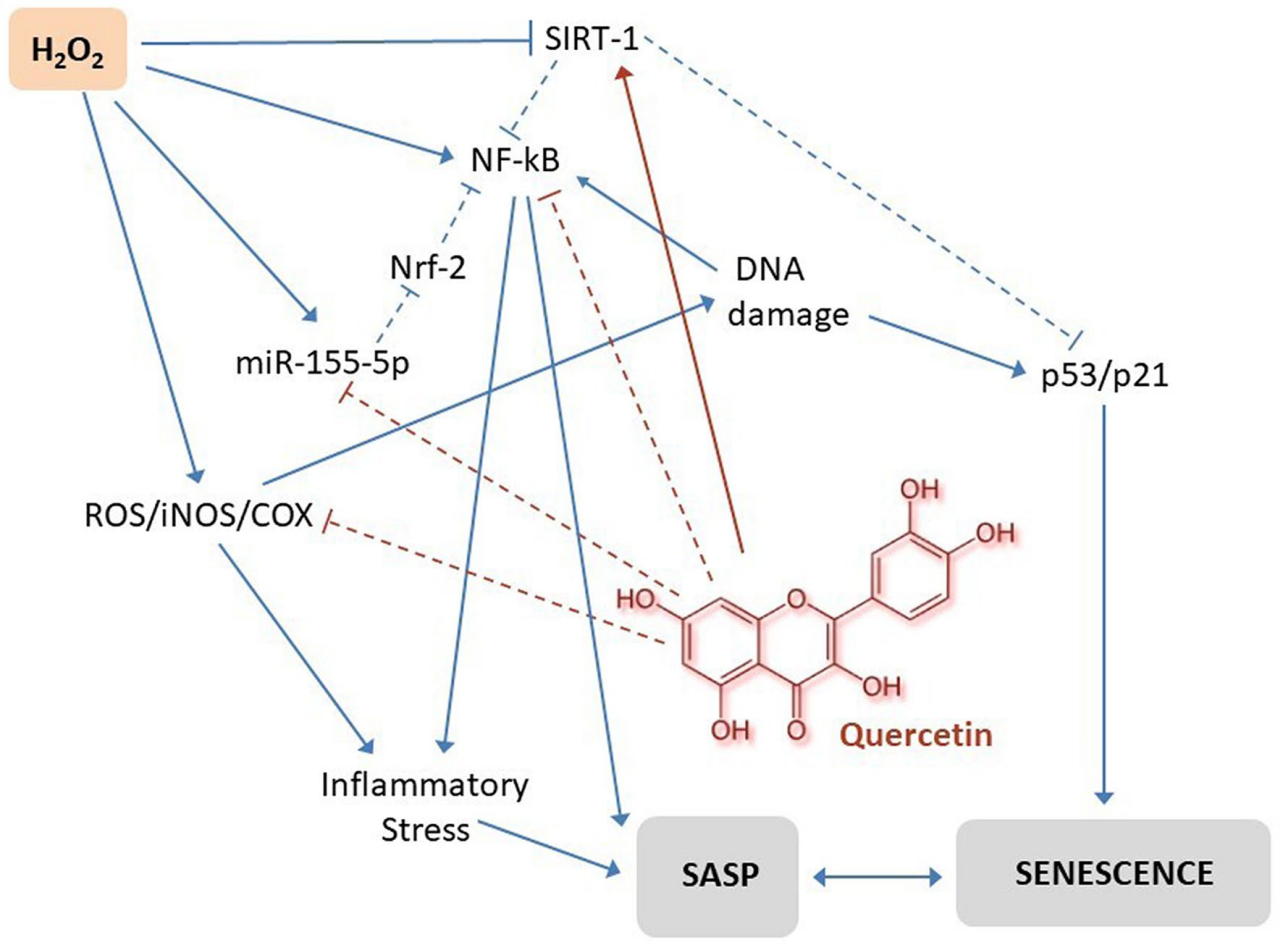
Hypothetical effects of Quercetin on pathways involved in AT cellular senescence. Quercetin activates SIRT1 that is involved in the regulation of cellular senescence by the activation of p53-p21 pathway. NF-kB, a major inducer of cellular senescence and appearance of SASP is in turn down-regulated indirectly by the inhibition of miR-155-5p affected from Quercetin. Intracellular ROS formation is also suppressed by Quercetin limiting its contribution to the cellular aging. The solid arrows represent the activation from one element to another and the dashed arrows indicate the inhibition.

It has been found that miR-155-5p could down-regulate the factor erythroid-derived 2, like 2 (Nrf2), a transcription factor of the family of basic leucine zipper proteins which modulates other genes involved in the regulation of the inflammatory response^45^. Thus, it is possible to hypothesize that an increase in miR-155-5p, induced by aging or by oxidative stress, could inhibit Nrf2 allowing the expression of NF-κB and the consequent increase of ROS and SASP (Fig. 7). Through dampening miR-155-5p, quercetin could reestablish the production of Nrf2, thus acting as an anti-aging compound (Fig. 7).

Finally based on the in vitro data of our experiment it can be hypothesized that quercetin may also modulate SIRT-1 in adipose cells, with a possible increase in its expression and downstream modulation of the senescence pathways like p53 and p21 (Fig. 7). However further experiments are needed to confirm these preliminary data.

SIRT1 decreases in many tissues during ageing and is considered a sensor of metabolism and it has been proposed as a potential therapeutic target for the treatment of age-related diseases^46,47^. Moreover, SIRT1 regulates AT metabolism affecting adipogenesis^48^, lipolysis and cellular metabolism^49^. It has been shown that SIRT1 contribute to maintain a physiological AT also through its anti-inflammatory abilities^50^. All these characteristics confer to SIRT-1 the potential to be a target of the senolyic and anti-inflammatory effects of quercetin.

In conclusion, some limits of the present study should be acknowledged, first of all the in vitro design of the study itself. In fact AT is composed of different cell types and the paracrine and endocrine hormonal milieu cannot be fully explored in an in vitro model. However the effects of a senolytic compound could be better explored in a controlled model of adipocyte and pre-adipocytes aging, in order to try to better characterized the mechanisms of action and the pathways involved. Second the use of only one model of induced senescence based on a protocol of repeated H_2_O_2_ treatments even though previously shown to induce senescence in vitro^30^; it would be interesting to test the effects of quercetin also in other senescent models as in vitro prolonged culture of 3T3-L1 adipocytes. To better explore the activation of NF-kB we should had performed assays on nuclear and cytoplasmatic fractions of the cells, evaluating p65 or measuring the DNA-binding activity of the NF-kB complex in nuclear extracts, by radiolabeling techniques. Finally, we cannot demonstrate a direct cause-effect relationship but only associations; further experiments, based on these data, should be performed to confirm for example the modulation of miR-155-5p by quercetin.

Our results all together confirm for quercetin a senolytic and anti-inflammatory role not only in senescent adipocytes but also for senescent pre-adipocytes; miR-155-5p inhibition and modulation could be among the targets of quercetin action in adipose cells.

## Supplementary Information

Supplementary Figures.
