# Supplementary figures and images for: Senolytic effects of quercetin in an in vitro model of pre-adipocytes and adipocytes induced senescence
Source: Sci Rep. 2021 Dec 1;11:23237. doi: 10.1038/s41598-021-02544-0 (PMC8636588; doi:10.1038/s41598-021-02544-0)

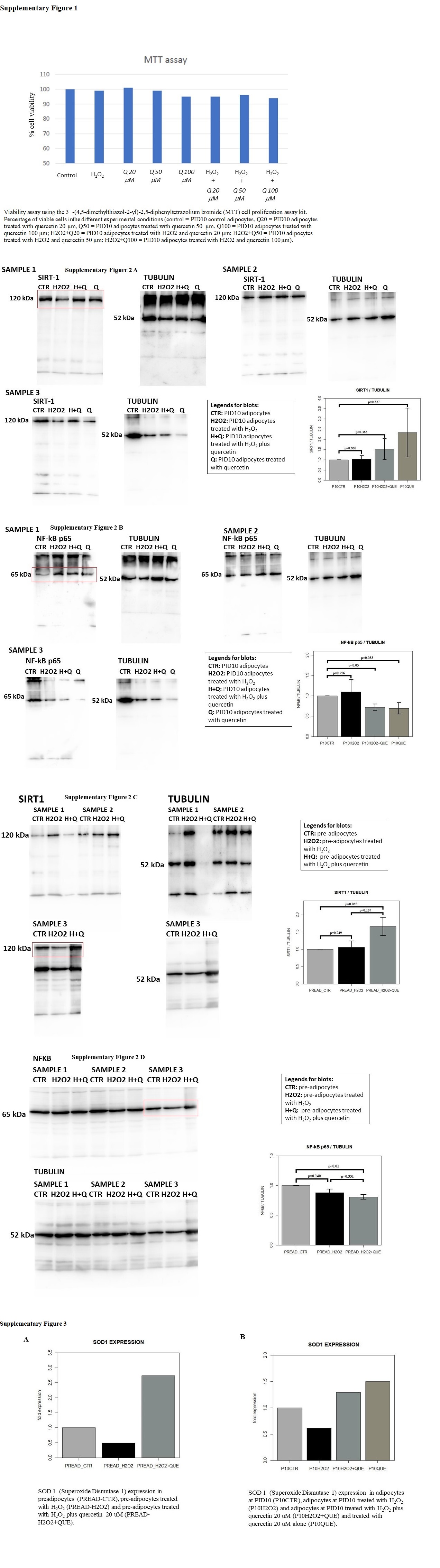

Supplement: Supplementary file 1 — Supplementary Figures. [file 41598_2021_2544_MOESM1_ESM.jpg]
